# Chemokine receptor CXCR2 in primary sensory neurons of trigeminal ganglion mediates orofacial itch

**DOI:** 10.3389/fnmol.2023.1279237

**Published:** 2023-10-26

**Authors:** Dong-Jin Li, Zhen-Juan Zhong, Xiao-Liang Wang, Na Wei, Si-Jia Zhao, Ting-Ting Shan, Ya-Ping Liu, Yao-Qing Yu

**Affiliations:** ^1^College of Life Sciences, Northwest University, Xi’an, China; ^2^Institute for Biomedical Sciences of Pain, Tangdu Hospital, Air Force Medical University, Xi’an, China; ^3^Key Laboratory of Brain Stress and Behavior, People’s Liberation Army, Xi’an, China

**Keywords:** CXCR2, trigeminal ganglion, orofacial itch, incision, virus

## Abstract

The CXCR2 chemokine receptor is known to have a significant impact on the initiation and control of inflammatory processes. However, its specific involvement in the sensation of itch is not yet fully understood. In this study, we aimed to elucidate the function of CXCR2 in the trigeminal ganglion (TG) by utilizing orofacial itch models induced by incision, chloroquine (CQ), and histamine. Our results revealed a significant up-regulation of CXCR2 mRNA and protein expressions in the primary sensory neurons of TG in response to itch stimuli. The CXCR2 inhibitor SB225002 resulted in notable decrease in CXCR2 protein expression and reduction in scratch behaviors. Distal infraorbital nerve (DION) microinjection of a specific shRNA virus inhibited CXCR2 expression in TG neurons and reversed itch behaviors. Additionally, the administration of the PI3K inhibitor LY294002 resulted in a decrease in the expressions of p-Akt, Akt, and CXCR2 in TG neurons, thereby mitigating pruritic behaviors. Collectively, we report that CXCR2 in the primary sensory neurons of trigeminal ganglion contributes to orofacial itch through the PI3K/Akt signaling pathway. These observations highlight the potential of molecules involved in the regulation of CXCR2 as viable therapeutic targets for the treatment of itch.

## Introduction

Itching is an unpleasant sensation that provokes the desire to scratch (Stander et al., 2003; Cevikbas and Lerner, 2020). Pruritic stimuli-induced hindlimb scratch was an indicator of itch, and forelimb wipe was an indicator of pain (Shimada and LaMotte, 2008). Cheek injections of pruritogens and algogens have proven reliable in confirming itch and pain. For instance, chloroquine (CQ) and histamine primarily induce scratching behaviors, while not eliciting significant wiping behaviors (Akiyama et al., 2010; Klein et al., 2011). β2-Microglobulin (β2-MG) and miR-711 cheek injections elicited scratching behaviors (Andoh et al., 2017; Han et al., 2018). Facial injection of lysophosphatidic acid has been found to induce itch behaviors (Kittaka et al., 2017). Robust scratching responses have also been observed following intradermal injection of compound 48/80 or HTMT (Zhu et al., 2020). Studies have shed light on the involvement of IL-13 receptor and IL-20 in facial itch and atopic dermatitis (Xiao et al., 2021; Lu et al., 2022). In a recent study, researchers utilized an experimental mouse model of nape wound healing and investigated the roles of TGF-β and IL-31 in itch (Xu et al., 2020). Given the existing knowledge, it is intriguing to explore whether facial incision induces pruritus and to reveal the underlying mechanisms.

Trigeminal ganglion (TG) neurons are integral to the sensory response of the face, as established by previous research (Cho et al., 2020). The trigeminal nerve predominantly serves to innervate the facial region, contributing to both motor and sensory functions (Graff-Radford et al., 2015). Electrophysiological investigations have revealed that a majority of primary and higher-order trigeminal sensory neurons are activated by both pruritic and algesic stimuli (Akiyama et al., 2010; Klein et al., 2011). Notably, recent studies have shed light on the role of specific TG neuron subtypes, such as somatostatin and MrgprA3-expressing neurons, in mediating itch sensations and selective induction of scratching behaviors (Huang et al., 2018; Sharif et al., 2020). Gaining a comprehensive understanding of the cellular and molecular mechanisms in orofacial itch would open up possibilities for identifying potential therapeutic targets for itch treatment.

Chemokines are a class of small secreted proteins characterized by the presence of conserved cysteine residues within their amino acid sequences (Silva et al., 2017). These proteins can be categorized into different subfamilies based on the position of their N-terminal cysteine residues, which include CC, CXC, XC, and CX3C (van der Vorst et al., 2015). Chemokines are known to bind and activate various chemokine receptors. These receptors belong to the family of seven-transmembrane G protein-coupled receptors and are classified into subfamilies including CCR, CXCR, XCR, and CX3CR (Zhang et al., 2017). Chemokines and their receptors expressed in neurons of the dorsal root ganglia (DRG) play significant roles in itch and pain (Deftu et al., 2017; Jing et al., 2018; Su et al., 2020). Numerous studies have provided evidence supporting the involvement of chemokines and receptors in DRG neurons in the regulation of itch. For instance, CCL2/CCR2 signaling in DRG neurons has been shown to be activated in contact hypersensitivity and contribute to the development of itch behavior (Jiang et al., 2019). CXCL1 and CXCL2, which act specifically through CXCR2, activate a sub-population of DRG neutrons and are associated with itch sensations (Deftu et al., 2017). Additionally, CXCL10 and its receptor CXCR3 have been found to be upregulated in the DRG and spinal cord and contribute to the development of chronic itch (Qu et al., 2015; Jing et al., 2018; Walsh et al., 2019). Existing evidence suggests that CXCL12 and its receptor CXCR4 in TG are involved in allergic contact dermatitis, and blockade of this signaling pathway alleviates itch sensations (Su et al., 2020). However, the understanding of chemokines and receptors in TG neurons is currently limited.

The CXCR2 chemokine receptor plays pivotal roles in initiating and regulating inflammatory responses and modulating neuronal signaling (Piotrowska et al., 2021). Upon activation, CXCR2 can initiate signal transduction through the phosphatidylinositol-4, 5-bisphosphate 3-kinase (PI3K) protein kinase B (PKB)/Akt pathway (Korbecki et al., 2022). Additionally, the expression and function of CXCR2 are subject to regulation by various intracellular signaling pathways, including the AMP-activated protein kinase, protein kinase, and extracellular signal-regulated kinase pathways (Yang et al., 2022). Studies have reported the involvement of CXCR2 in the maintenance of neuropathic pain and inflammatory pain (Gao and Ji, 2010; Cao et al., 2016; Xu et al., 2017). However, the specific role of CXCR2 in TG neurons in the context of itch sensation remains largely unexplored.

In the present study, we aimed to elucidate the roles of chemokine receptor CXCR2 in the primary sensory neurons of the trigeminal ganglion in orofacial itch models induced by incision, chloroquine, and histamine. To achieve this, we employed a novel approach involving microinjection of the distal infraorbital nerve (DION) to assess the effects of chemical interventions on trigeminal sensory neurons. The functions of CXCR2 in orofacial itch were evaluated using a CXCR2 inhibitor and a specific shRNA virus. Additionally, we investigated the involvement of the PI3K/Akt signaling pathway in CXCR2 expression and orofacial itch behaviors. Our study highlighted the potential of molecules that regulate CXCR2 as promising therapeutic targets for the treatment of itch.

## Materials and methods

### Experimental animals

Adult male C57BL/6 mice (8 weeks) were prepared from Laboratory Animal Center of Air Force Medical University (AFMU). The animals had access to water and food *ad libitum* and were maintained at room temperature (22–26°C) with a light/dark cycle of 12 h. The number of animals used and their suffering were minimized. All animal experiments were carried out according to the ARRIVE guidelines and approved by the Institutional Animal Care and Use Committee of AFMU.

### Cheek itch models

The cheek incision itch model was modified as previously described (Xu et al., 2020). C57BL/6 mice were anesthetized with 2% isoflurane and the right sides of their cheeks were shaved. A 5-mm-long full-thickness cheek incision was made one the cheek. Scratch behavior was recorded at specific time points, including 6 h, day 1, day 3, and day 5. In line with our previous study (Yu et al., 2017), intradermal injections of chloroquine (CQ, 100 μg/20 μL, Sigma, USA, C6628) and histamine (100 μg/20 μL saline, Sigma, USA, H7125) were administered into the cheek. For the cheek model of itch discrimination, mice were placed in transparent plastic recording chambers, and their behaviors were recorded using video for subsequent offline analysis. Each scratching bout was defined as rapid brushing of the face by the hindpaw on the ipsilateral side. Scratching or wiping behaviors on the contralateral side were not included in the analysis.

### Drug delivery

Intrathecal (i.t.) and intraperitoneal (i.p.) delivery methods were previously described (Wei et al., 2022). CXCR2 antagonist SB225002 (5 mg/kg, Sigma, USA, SML0716) or saline was administered via i.p. delivery once daily for two consecutive days. PI3K inhibitor LY294002 (2 mM/10 μL, MCE, USA, HY-10108) was administered via i.t. delivery twice daily for three consecutive days. SB225002 and LY294002 were pre-injected 30 min before pruritic stimuli challenge.

### DION microinjection

Distal infraorbital nerve (DION) was prepared according to a previous report (Hardt et al., 2019). Briefly, the animal was anesthetized with 2% pentobarbital sodium, and a 5-mm-long skin incision was made below the infraorbital foramen. Then the distal infraorbital nerve (DION), which is a branch of the medial trunk of the trigeminal nerve, was then exposed for microinjection.

For the microinjection of the retrograde tracer into DION, 1, 1′-dioctadecyl-3, 3, 3′, 3′-retramethy-lindocarbo-cyanine perchlorate (DiI, Fluka, Japan) was dissolved in dimethylsulfoxide (DMSO) at a concentration of 170 mg/mL, and then diluted to 17 mg/mL with 0.9% sterile saline. To retrograde label TG neurons, a microinjection of 1 μL of DiI into DION was performed following the method described in our previous study (Yu et al., 2013).

For the DION virus microinjection, an adeno-associated virus serotype 9 (AAV9) encoding shRNA was designed based on mouse CXCR2 (NM_009909.3). The AAV scramble or CXCR2 shRNA virus, with a concentration of 1 × 10^12^ viral genomes (vg)/mL, was engineered to express the green fluorescence protein (GFP) (BrainVTA, China). The sequence for the scramble shRNA is CCTAAGGTTAAGTCGCCCTCG, and the sequence for the CXCR2 shRNA is GGGAGAATTCAAGGTGGATAA. A microsyringe was used to inject 1 μL of the scramble or CXCR2 shRNA virus into DION. The mice were allowed to recover for 3–4 weeks before conducting behavioral and other experiments.

### Real-time quantitative PCR

The total RNA of the TG was extracted using TRIzol reagent (Thermo Fisher Scientific). 1 ug of total RNA was reverse transcribed to cDNA using the Transcriptor First Strand cDNA Synthesis Kit (Roche, Switzerland, 04897030001). Quantitative polymerase chain reaction (qPCR) analysis was performed using a CFX96TM Real-Time system (Bio-Rad) with Power SYBR Green PCR Master Mix (Life Technologies). The following primers were used: *CXCL1* forward, 5′-TGGCTGGGATTCACCTCAAG-3′; *CXCL1* reverse, 5′-CCGTTACTTGGGGACACCTT-3′; *CXCR1* forward, 5′-CCAGCTGGTGCCTCAGATCAA-3′; *CXCR1* reverse, 5′-AAATAATCTCCAGTGGGCAGCA-3′; *CXCR2* forward, 5′-TCTGCTCACAAACAGCGTCGT A-3′; *CXCR2* reverse, 5′-GAGTGGCATGGGACAGCATC-3′; *CXCR3* forward, 5′-TACCTTGAGGTTAGTGAACGTCA-3′; *CXCR3* reverse, 5′-CGCTCTCGTTTTCCCCATAATC-3′; *CXCR4* forward, 5′-AGGAAACTGCTGGCTGAAAAGG-3′; *CXCR4* reverse, 5′-GGAATTGAAACACCACCATCCA-3′; *CXCR5* forward, 5′-TGGCCTTCT ACAGTAACAGCA-3′; *CXCR5* reverse, 5′-GCATGAATACCGCCTTAAAGGAC-3′; *CXCR6* forward, 5′-TCTGCCCTTTTGGGCCTATG-3′; *CXCR6* reverse, 5′-TTGAAGGCCTTGGTAGCCTG-3′; *GAPDH* forward, 5′-CCCAGCAAGGACACTGAGCAA-3′; *GAPDH* reverse, 5′-TTATGGGGGTCTGGGATGGAAA-3′. All primer pairs were designed for the same cycling conditions: 10 min at 95°C for initial denaturation, 40 cycles of 10 s at 95°C for denaturation, 15 s at 61.2°C for annealing, and 20 s at 72°C for extension.

### Immunohistochemistry staining

Immunohistochemistry staining was performed as previously described (Wei et al., 2022). TGs were dissected, postfixed for 8 h, and cryoprotected in 30% sucrose in PBS overnight at 4°C. Transverse frozen sections (20 μm thick) were cut on a CM1900 freezing microtome (Leica, Germany), incubated for 4 h in 0.05% Triton X-100 and 2% donkey serum in phosphate buffered saline (PBS) at room temperature, and incubated with primary antibodies at 4°C overnight with agitation. After three washes with PBST, the sections were incubated with secondary antibodies for 3 h at room temperature. The following primary antibodies were used: rabbit anti-CXCR2 (1:100, Boster, USA, A00455S347), chicken anti-GFP (1:500, Abcam, USA, ab13970), mouse anti-Akt (1:200, Proteintech, USA, 60203-2-Ig). The secondary antibodies were Cy3 Donkey Anti-rabbit IgG (1:500, Jackson ImmunoResearch, USA, 711-165-152), Alexa Fluor 488 Donkey Anti-chicken IgG (1:500, Jackson ImmunoResearch, USA, 703-545-155). Alexa Fluor 488 Donkey Anti-mouse IgG (1:500, Jackson ImmunoResearch, USA, 715-545-151) and lectin from Bandeiraea simplicifolia BS-I isolectin B4 FITC conjugate (1:100, Sigma, USA, L2895). The nuclei were stained with DAPI (1:2,000, Sigma, USA, D9542). Photomicrographic images were obtained under a laser scan confocal fluorescent microscope (Olympus FV1000, Japan). Images were analyzed by Image-Pro Plus digitizing software (Olympus, Japan).

### Western blot

Western blotting was performed as previously described (Wei et al., 2022). Total proteins from mice TGs were extracted by homogenization in ice-cold RIPA lysis buffer (Applygen Technologies, China). Membrane proteins and cytoplasmic proteins from mice TGs were extracted by homogenization. Protein concentrations were determined by using a BCA™ protein assay kit (Thermo Scientific, USA). The samples were heated for 10 min at 95°C with SDS-PAGE sample buffer, and the same amounts of proteins (50 μg) were separated by 10% SDS-PAGE separation gels and subsequently transblotted onto PVDF membranes (Immobilon P, Millipore, Billerica, MA). We used rabbit anti-CXCR2 (phosphor-IL8 Beta) (1:1,000, Boster, USA, A00455S347), mouse anti-p-Akt1/2/3 antibody (B-12) (1:500, Santa Cruz Biotechnology, USA, sc-377556) and mouse anti-Akt1 Antibody (B-1) (1:500, Santa Cruz Biotechnology, USA, sc-5298) as primary antibodies and goat anti-rabbit IgG conjugated to horseradish peroxidase (HRP) and goat anti-mouse IgG conjugated to HRP (1:2,000, ZSGB-Bio, Beijing, China) as the secondary antibodies. Mouse anti-GAPDH antibody (1:2,000, Proteintech, USA, 60004-1-Ig) was used as an internal control.

### Statistical analysis

For behavioral experiments, *n* refers to the number of animals. For immunohistochemistry imaging data, the number of animals and sections used is indicated in the legend. Statistical analyses were performed using GraphPad Prism software. All data are expressed as mean ± standard error (SEM) unless otherwise specified. Descriptions of the tests used and n are located in the figure legends. Significance was defined as follows: *p < 0.05, **p < 0.01, and ***p < 0.001.

## Results

### The expressions of *CXCRs* mRNA in TGs with orofacial itch

**Figure 1:**
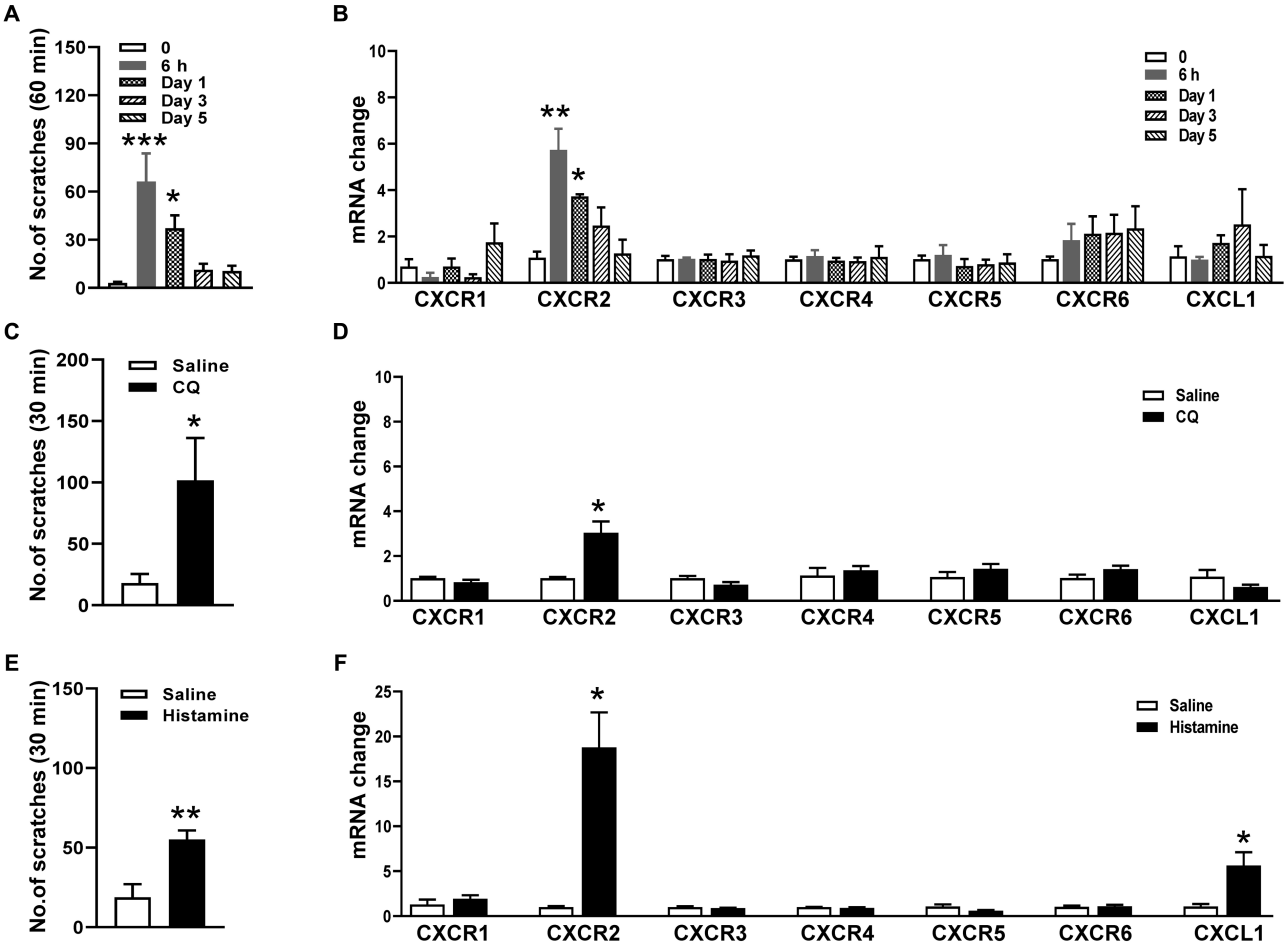
Expressions of *CXCRs* mRNA in TG in orofacial itch. **(A)** Mean number of scratches at individual time points 0, 6 h, day 1, day 3, and day 5 after incision (*n* = 6 mice for each group). **(B)** The mRNA expressions of *CXCL1* and *CXCR1-CXCR6* in TG at 6 h, day 1, day 3, and day 5 after incision (*n* = 3 mice for each group). **(C)** Mean number of scratches of mice after saline and CQ injection on the right face (*n* = 5 mice for each group). **(D)** The mRNA expressions of *CXCL1* and *CXCR1-CXCR6* after injection of saline and CQ (*n* = 3–4 mice for each group). **(E)** Mean number of scratches of mice after saline and histamine injection on the right face (*n* = 6 mice for each group). **(F)** The mRNA expressions of *CXCL1* and *CXCR1-CXCR6* after saline and histamine injection (*n* = 3–4 mice for each group). One-way ANOVA followed by Dunnett’s multiple comparisons test in **(A,B)**, and unpaired *t* test in **(C–F)**. * *p* < 0.05, ** *p* < 0.01, *** *p* < 0.001. Error bars represent the mean ± SEM.

To determine whether CXCR2 in TG is involved in orofacial itch, we establish a cheek incision itch model and examine scratch behaviors at individual time points 0, 6 h, day 1, day 3, and day 5. The scratching behaviors increased significantly at 6 h (3 ± 1 versus 66 ± 18, *p* < 0.001) and day 1 (3 ± 1 versus 37 ± 8, *p* < 0.05, Figure 1A). qPCR was used to examine the mRNA expressions of chemokine *CXCL1* and six chemokines receptors (*CXCR1, CXCR2, CXCR3, CXCR4, CXCR5, CXCR6*) in TG during cheek incision itch. *CXCR2* mRNA expression was up-regulated at 6 h (1.1 ± 0.3 versus 5.7 ± 0.9, *p* < 0.001) and day 1 (1.1 ± 0.3 versus 3.7 ± 0.1, *p* < 0.05, Figure 1B). Compared to control, CQ and histamine injections in the cheek evoked itch behavior (18 ± 7 versus 102 ± 34 and 19 ± 8 versus 55 ± 6, *p* < 0.05 and *p* < 0.01, Figures 1C,E). *CXCR2* mRNA expression was significantly increased within 1 h after CQ (1.0 ± 0.1 versus 3.0 ± 0.5, *p* < 0.05, Figure 1D) and histamine (1.0 ± 0.1 versus 18.8 ± 3.9, *p* < 0.05, Figure 1F). These results indicated that the *CXCR2* gene was up-regulated in TG under itch conditions.

### Upregulation of CXCR2 protein expression in TGs with orofacial itch

**Figure 2:**
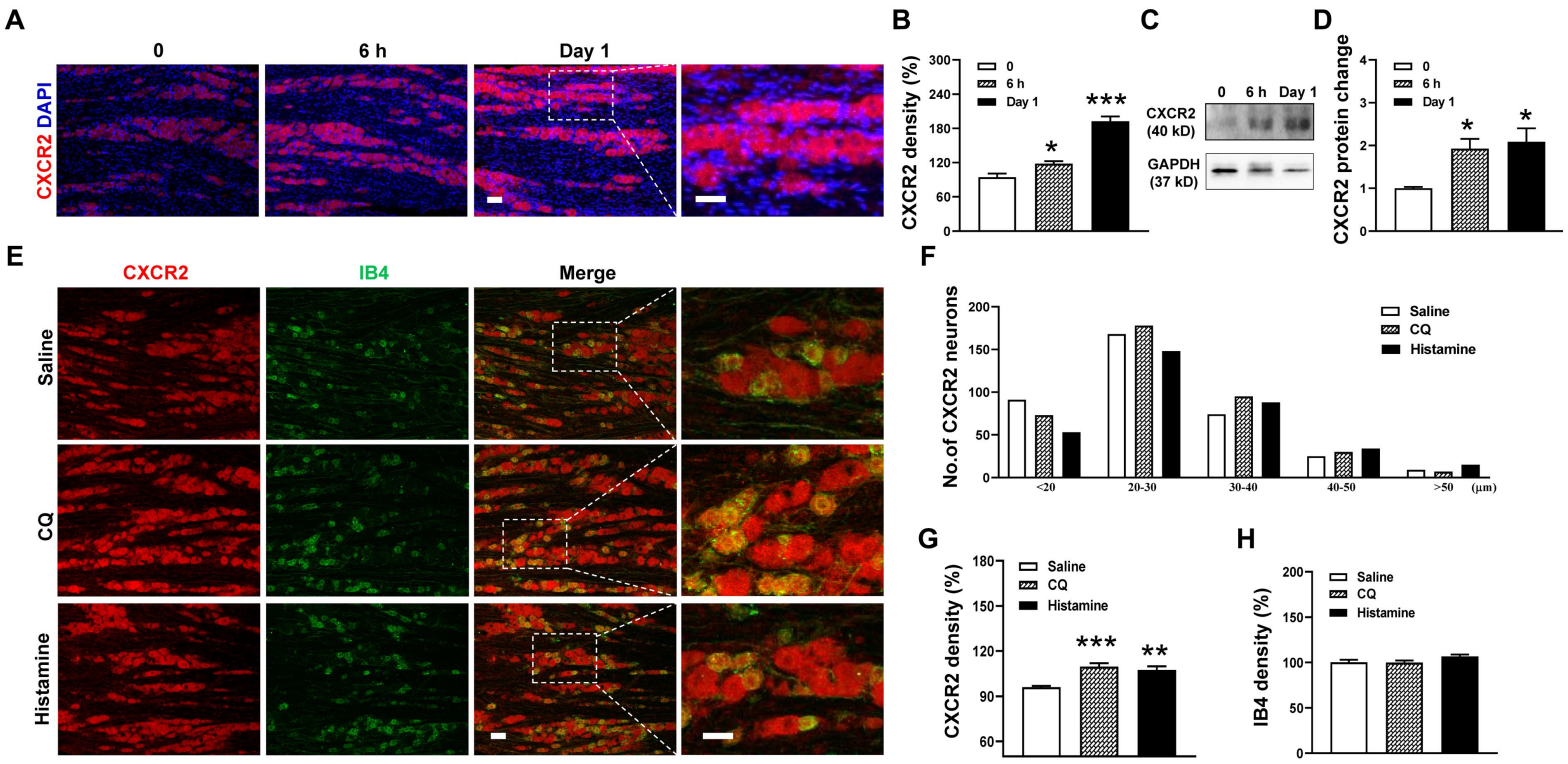
Upregulation of the expression of CXCR2 protein in TG in orofacial itch. **(A)** Double immunohistochemistry of CXCR2 and DAPI at 6 h and day 1 after incision. **(B)** Statistical analysis of CXCR2 immunofluorescence density at 6 h and day 1 after incision (*n* = 3 mice and 8–9 sections for each group). **(C)** Representative Western blot of CXCR2 in TG with incision. **(D)** Statistical analysis of CXCR2 protein with incision (*n* = 3 mice for each group) **(E)** Double immunohistochemistry of CXCR2 and IB4 with CQ and histamine injection. **(F)** Mean number of CXCR2 neurons after CQ and histamine injection (*n* = 3 mice and 6–8 sections for each group). Scale bars, 50 μm. One-way ANOVA followed by Dunnett’s multiple comparisons test in **(B,D,G,H)**. * *p* < 0.05, ** *p* < 0.01, *** *p* < 0.001. Error bars represent the mean ± SEM.

Based on the change in *CXCR2* mRNA expression, we performed immunohistochemistry and Western blot of CXCR2 in TGs. Compared to the control, the immunofluorescence density of CXCR2-positive staining increased at 6 h (94.5 ± 6.3% versus 118.1 ± 4.4%, *p* < 0.05) and day 1 after incision (94.5 ± 6.3% versus 192.7 ± 8.2%, *p* < 0.001, Figures 2A,B). Western blot further confirmed that the expression of CXCR2 protein was significantly increased in the same time points (1.0 ± 0.03 versus 1.9 ± 0.2 and 2.1 ± 0.3, *p* < 0.05, Figures 2C,D). Then, we examined the cellular localization of CXCR2 by double immunofluorescent labeling with IB4, a small-sized neuron marker, after CQ or histamine injection (Figure 2E). We counted the distribution of CXCR2-positive staining in the different size of TG neurons from saline (367 neurons), CQ (383 neurons), and histamine (338 neurons) treatments. The results indicated that the CXCR2-positive neurons widely distributed in small, medium, and large neurons in TGs (Figure 2F). Immunohistochemistry data analysis revealed that the CXCR2 immunofluorescence density was increased by CQ and histamine (96.0 ± 0.9% versus 109.8 ± 2.2% and 107.4 ± 2.4%, *p* < 0.001 and *p* < 0.01, Figure 2G). The immunofluorescence density of IB4 was not changed by CQ and histamine (Figure 2H). These results led us to examine the biological function of CXCR2 in TG neurons for itch.

### Effects of CXCR2 antagonist on orofacial itch

**Figure 3:**
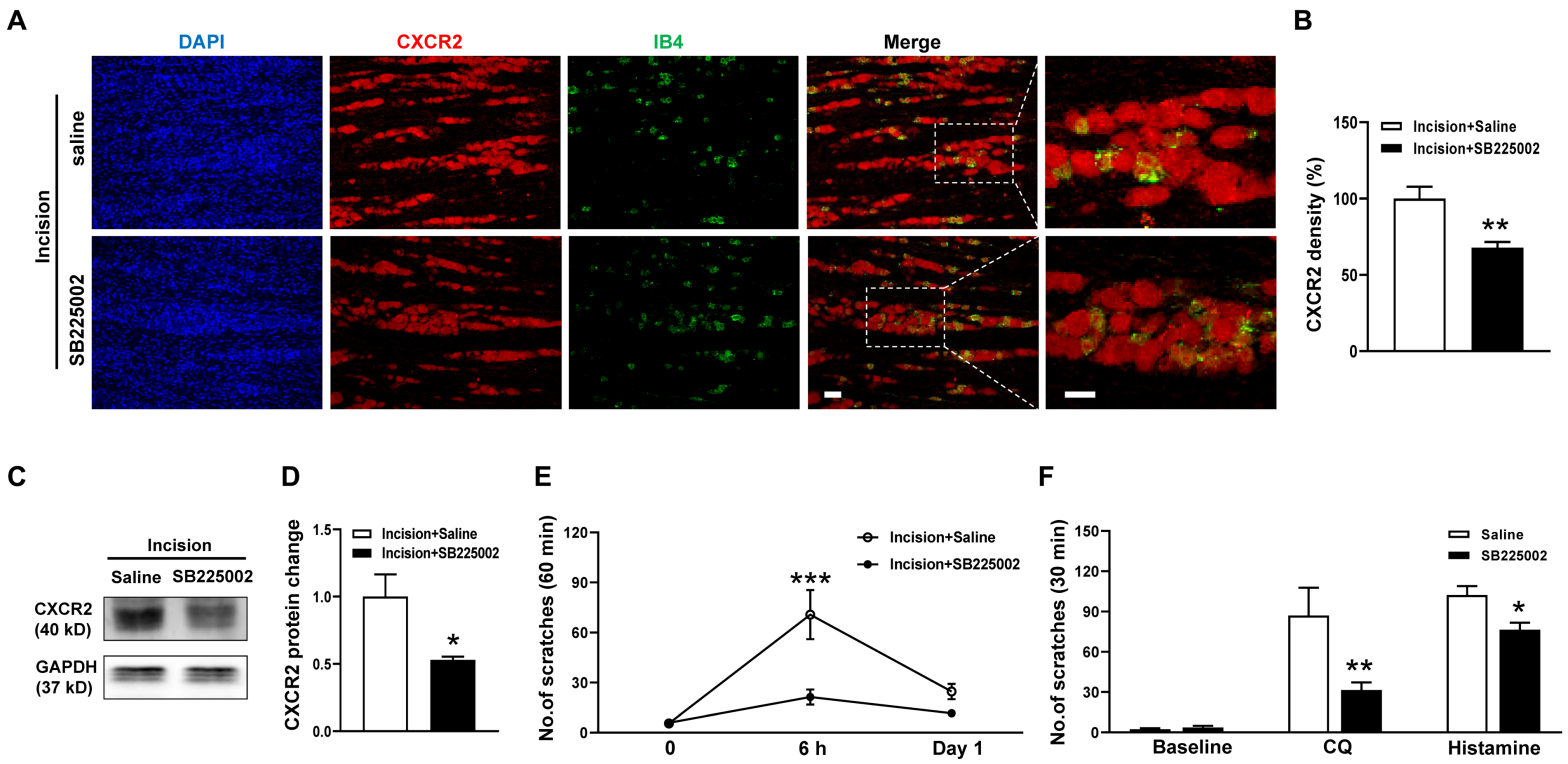
Effects of the CXCR2 Antagonist SB225002 on oroficial itch. **(A)** Double immunohistochemistry of CXCR2 and IB4 in the saline and SB225002 treatment groups. **(B)** Statistical analysis of the immunofluorescence density of CXCR2 in saline and SB225002 treatment (*n* = 3 mice and 11 sections for each group). **(C)** Representative Western blot of CXCR2 with saline and SB225002 after incision. **(D)** Statistical analysis of CXCR2 protein with saline or SB225002 after incision (*n* = 3 mice for each group). **(E)** Effects of SB225002 on scratch behaviors induced by incision (*n* = 10 mice for each group). **(F)** Effects of SB225002 on scratch behaviors induced by CQ and histamine (*n* = 8 mice for each group). Scale bars, 50 μm. Unpaired t test in **(B,D,F)** and 2-way ANOVA in **(E)**. * *p* < 0.05, ** *p* < 0.01, *** *p* < 0.001. Error bars represent the mean ± SEM.

To dissect the functional contribution of CXCR2 to orofacial itch, we intraperitoneally (i.p.) administered the CXCR2 antagonist SB225002. The immunohistochemistry results showed that SB225002 significantly reduced the immunofluorescence density of CXCR2 in TG (100.0 ± 7.8% versus 67.8 ± 3.8%, *p* < 0.001, Figures 3A,B). Western blot confirmed the suppressive effects of SB225002 on the expression of CXCR2 protein (1.0 ± 0.2 versus 0.5 ± 0.02, *p* < 0.05, Figures 3C,D). Incision itch behaviors were attenuated by SB225002 at 6 h (71 ± 15 versus 21 ± 5, *p* < 0.001) (Figure 3E). Additionally, the CXCR2 antagonist SB225002 significantly inhibited scratching behaviors induced by CQ (87 ± 21 versus 32 ± 6, *p* < 0.01) and histamine (102 ± 7 versus 76 ± 5, *p* < 0.05) (Figure 3F). These results supported the crucial role of CXCR2 in TG neurons, and its block would reverse orofacial itch behaviors.

### Effects of CXCR2 shRNA on orofacial itch

**Figure 4:**
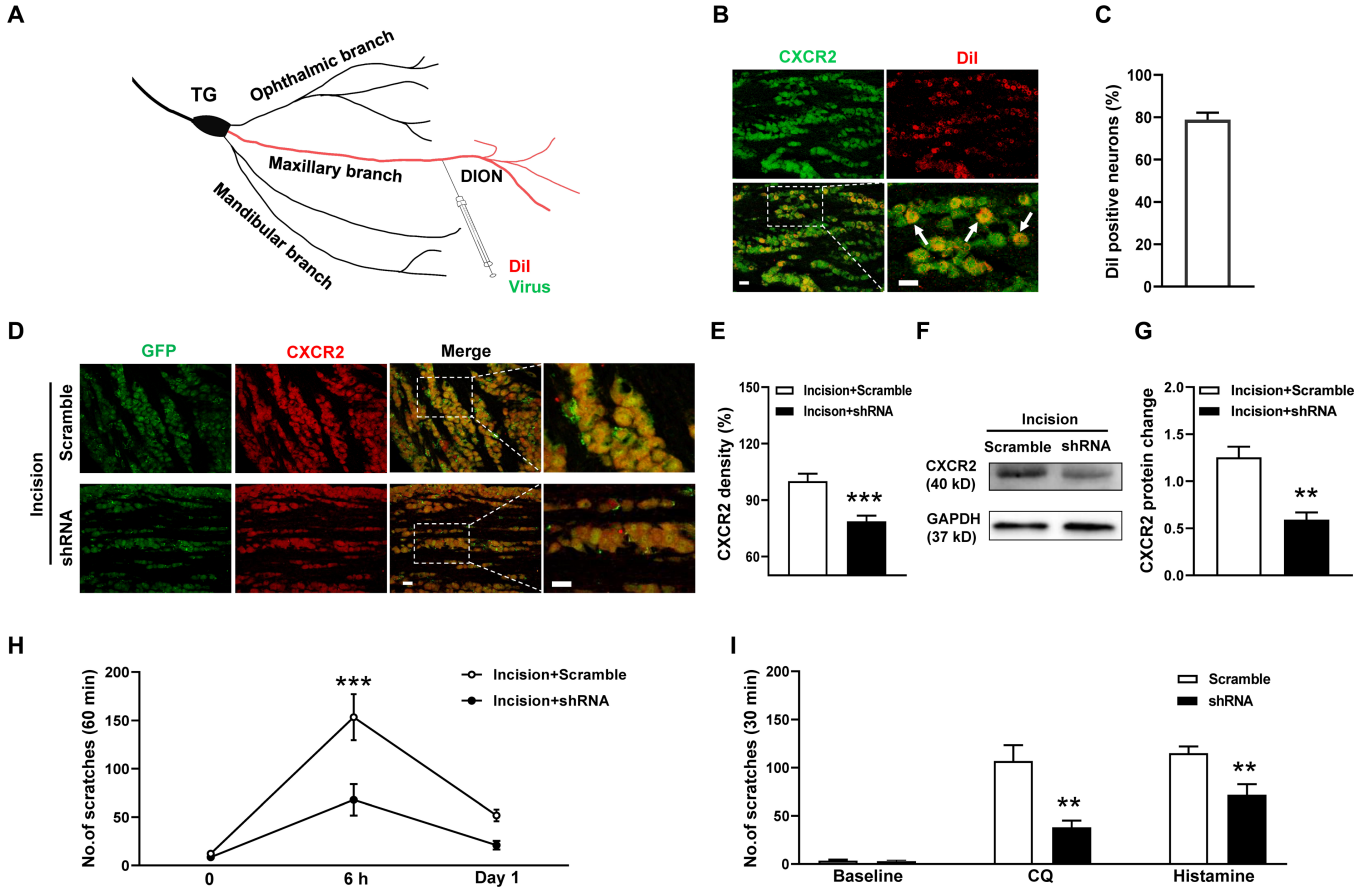
DION microinjection and effects of CXCR2 shRNA on orofacial itch. **(A)** Schematic of DiI and shRNA virus microinjection into DION. **(B)** Double immunohistochemistry of CXCR2 and DiI in TG neurons (*n* = 3 mice and 10 sections). Arrows indicate DiI-labeled CXCR2 neurons. **(C)** The proportion of DiI-positive neurons in TG neurons (*n* = 3 mice and 10 sections for each group). **(D)** Examples of immunofuorescence images of CXCR2 and GFP treated with scramble and shRNA. **(E)** Statistical analysis of CXCR2 immunofluorescence density with scramble and shRNA (*n* = 3 mice and 9 sections for each group). **(F)** Representative Western blot of CXCR2 with scramble and shRNA. **(G)** Statistical analysis of CXCR2 protein in TG with scramble and shRNA (*n* = 3 mice for each group). **(H)** Effects of CXCR2 shRNA injection on incision-induced scratch behaviors (*n* = 5–6 mice for each group). **(I)** Effects of the CXCR2 shRNA virus on scratch behaviors induced by CQ and histamine (*n* = 6–7 mice for each group). Scale bars, 50 μm. Unpaired t test in **(E,G,I)** and 2-way ANOVA in **(H)**. * *p* < 0.05, ** *p* < 0.01, *** *p* < 0.001. Error bars represent the mean ± SEM.

To specifically inhibit CXCR2, we generated the CXCR2 shRNA virus and transmitted the virus into TGs using a distal infraorbital nerve (DION) microinjection approach (Figure 4A). We used the retrograde tracer DiI to examine the efficiency of DION microinjection. The immunostaining result revealed that DiI labeled 79% of CXCR2-positive TG neurons (Figures 4B,C). By DION microinjection approach, we further confirmed the high transfection efficiency of the virus (Figure 4D). The immunostaining results showed that the immunofluorescence density of CXCR2 in TG was significantly decreased by shRNA virus (100.0 ± 4.0% versus 78.7 ± 3.1%, *p* < 0.001, Figure 4E). Western blot confirmed the inhibitory effect of the CXCR2 shRNA virus (1.2 ± 0.1 versus 0.6 ± 0.1, *p* < 0.01, Figures 4F,G). Behavioral assays indicated that CXCR2 shRNA treatment suppressed incision-induced scratches at 6 h (153 ± 24 versus 68 ± 16, *p* < 0.001, Figure 4H). In cheek itch models, we revealed that the CXCR2 shRNA virus caused a significant reduction in itching responses induced by CQ (107 ± 17 versus 38 ± 7, *p* < 0.01) and histamine (115 ± 7 versus 72 ± 11, *p* < 0.01) (Figure 4I). These results verified the viral transduction efficiency of CXCR2 shRNA in TG neurons and DION microinjection could provide a novel strategy in modulating orofacial itch.

### Effects of PI3K/Akt signal pathway on CXCR2 expression and orofacial itch

**Figure 5:**
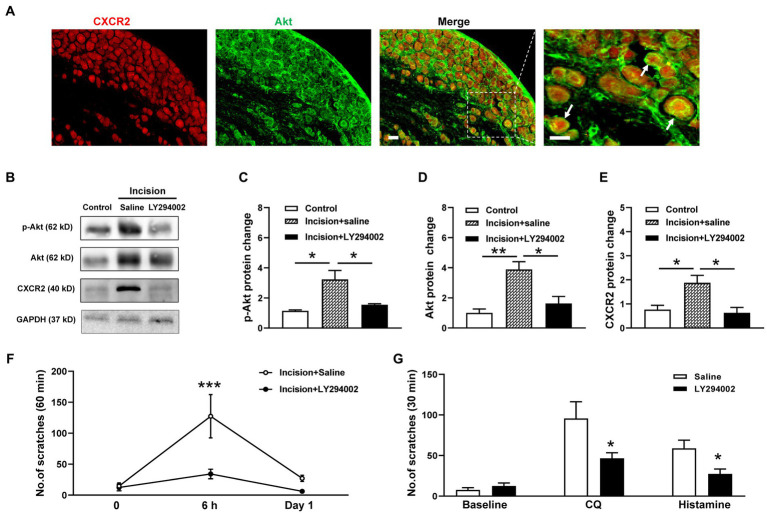
Effects of the CXCR2 and PI3K/Akt signal pathway on orofacial itch. **(A)** Double immunohistochemistry of CXCR2 and Akt in TG. Arrows indicate CXCR2 and Akt positive neurons. **(B)** Representative Western blot of p-Akt, Akt, and CXCR2 with saline and LY294002 after incision. **(C)** Statistical analysis of the p-Akt protein with saline and LY294002 after incision (*n* = 3 mice for each group). **(D)** Statistical analysis of Akt protein with saline and LY294002 after incision (*n* = 4 mice for each group). **(E)** Statistical analysis of the CXCR2 protein with saline and LY294002 after incision (*n* = 3 mice for each group). **(F)** Effects of LY294002 on incision-induced scratch behavior (*n* = 5 mice for each group). **(G)** Effects of LY294002 on incision-induced scratch behaviors (*n* = 7 mice for each group). Scale bars, 50 μm. One-way ANOVA followed by Tukey’s multiple comparisons test in **(C,D,E)**, 2-way ANOVA in **(F)**, and unpaired t test in **(G)**. * *p* < 0.05, ** *p* < 0.01. Error bars represent the mean ± SEM.

Then we investigate the potential roles of PI3K/Akt signal pathway in CXCR2-mediated itch. Coexpressions of CXCR2 and Akt in primary sensory neurons of TG were validated by double immunofluorescent staining (Figure 5A). Western blot showed that the PI3K inhibitor LY294002 attenuated incision-evoked upregulations of p-Akt (1.1 ± 0.1 versus 3.2 ± 0.6 versus 1.5 ± 0.1, *p* < 0.05, Figures 5B,C), Akt (1.0 ± 0.3 versus 3.9 ± 0.5 versus 1.6 ± 0.5, *p* < 0.01 and *p* < 0.05, Figures 5B,D), and CXCR2 (0.8 ± 0.2 versus 1.9 ± 0.3 versus 0.6 ± 0.2, *p* < 0.05, Figures 5B,E). Furthermore, LY294002 dramatically inhibited incision itch behaviors at 6 h (127 ± 35 versus 30 ± 8, *p* < 0.001) (Figure 5F). Additionally, PI3K inhibitor LY294002 suppressed scratching behaviors induced by CQ (96 ± 21 versus 47 ± 7, *p* < 0.05) and histamine (59 ± 10 versus 27 ± 6, *p* < 0.05) (Figure 5G). Taken together, these results suggested that PI3K/Akt signal pathway was involved in the expression of CXCR2 and contributed to modulation of orofacial itch.

## Discussion

The trigeminal ganglion (TG) is responsible for the sensory response of the face (Cho et al., 2020). The cellular and molecular mechanisms of orofacial itch mediated by TG neurons are complex. Previous studies have shown that endothelin receptor (Gomes et al., 2012) and transient receptor potential ion channel A1 (Wilson et al., 2011; Liu et al., 2021) play roles in mediating pruritus. Additionally, somatostatin or MrgprA3 neurons in TG have been found to be pruriceptors that mediate scratching behaviors (Huang et al., 2018; Sharif et al., 2020). The endogenous lipid mediator resolvin D3 has been shown to reduce TRPV1-dependent acute itch and chronic psoriasis itch (Lee et al., 2020). In the present study, we reveal the novel contribution of CXCR2 in TG neurons for itch modulation.

CXCR2 is well known for its involvement in various inflammatory diseases. For examples, the CXCL1/CXCR2 axis has been implicated in colon cancer metastasis and gastric cancer progression (Cheng et al., 2019). CXCR2 activation has also been linked to nociceptive behavior, bladder inflammation, and endothelial cell function (Singh et al., 2011; Dornelles et al., 2014). In terms of somatosensory modulation, CXCR2 has been associated with neuropathic pain and inflammatory pain (Gao and Ji, 2010; Cao et al., 2016; Xu et al., 2017). In our study, we found that CXCR2 mRNA and protein levels were upregulated in TG neurons during orofacial itch modulation induced by incision, CQ, and histamine. Importantly, inhibition of CXCR2 expression using an inhibitor and shRNA virus reversed the itch behaviors. These findings suggest that molecules targeting CXCR2 could be promising therapeutic targets for itch treatment.

When CXCL1 binds to CXCR2 on the cell surface, the associated G protein is activated, leading to the activation of PI3K/Akt and RAS–ERK signal pathways (Cheng et al., 2019). In the present study, we showed that the PI3K inhibitor LY294002 suppressed the expression of CXCR2 in TG and scratching behaviors, indicating that the PI3K/Akt signal pathway modulated CXCR2 expression and orofacial itch. However, whether the PI3K/Akt signal pathway affects CXCR2 membrane trafficking or gene expression remains an exciting question. Future experiments might investigate the intracellular mechanism of CXCR2 expressions in TG neurons.

Interventions in the trigeminal somatosensory system offer potential therapeutic options for orofacial diseases. Peripheral nerve neuromodulation techniques, such as invasive epidural cortical stimulation and peripheral surgical neurectomy, have shown promise in alleviating neuropathic pain (Wei and Jensen, 2018). In addition to these approaches, phenol/glycerol injections into the trigeminal nerve have been explored as an alternative therapeutic option (Wilkinson, 1999). Repetitive transcranial magnetic stimulation (rTMS) is a relatively new technology that offers the possibility of assessing the responsiveness of patients with trigeminal neuropathic pain to invasive epidural cortical stimulation (Obermann, 2019). A subgroup of highly disabled patients with trigeminal autonomic cephalalgias have not responded well to drug treatments and have to sought alternative options, including injectable treatments, nerve blocks, and various surgical procedures. Unfortunately, these alternative treatments, such as microvascular decompression, glycerol rhizotomy, trigeminal nerve radiofrequency ablation, balloon compression of the Gasserian ganglion, and Gamma knife therapy, have had limited success, with the adverse effects outweighing the benefits (Miller and Matharu, 2014). Therefore, further evaluation is needed to determine the overall efficacy of interventions in the trigeminal somatosensory system.

Directly controlling the activity of trigeminal ganglion (TG) neuronal soma is a complex and challenging procedure. For instance, a paramedian neck incision is made to visualize the trigeminal nerve by exposing and opening the tympanic bulla and tympanic cavity (Hara and Kobayashi, 1992). In another approach, a small hole is created in the skull without disturbing the meninges, and a guide cannula is implanted in the left TG (Shore et al., 2000; Han et al., 2012). Alternatively, a needle connected to a syringe is inserted medially into the zygomatic process through the infraorbital foramen at an angle of approximately 10° relative to the midline of the head. The needle then passes through the foramen rotundum and reaches the trigeminal ganglion (Gomes et al., 2018). In our study, we successfully manipulate CXCR2 expressions in TG neurons through DION microinjection. This approach manipulated a majority of CXCR2-positive TG neurons and effectively reduced CXCR2 expression and itch behaviors. DION microinjection holds promise as a novel therapeutic approach for orofacial itch and other intractable diseases.

## Data availability statement

The original contributions presented in the study are included in the article/supplementary material, further inquiries can be directed to the corresponding author.

## Ethics statement

The animal study was approved by Institutional Animal Care and Use Committee of AFMU. The study was conducted in accordance with the local legislation and institutional requirements.

## Author contributions

D-JL: Data curation, Formal analysis, Investigation, Methodology, Writing – original draft, Writing – review & editing. Z-JZ: Data curation, Investigation, Methodology, Writing – original draft. X-LW: Data curation, Investigation, Methodology, Writing – review & editing. NW: Data curation, Investigation, Methodology, Writing – review & editing. S-JZ: Data curation, Investigation, Methodology, Writing – review & editing. T-TS: Data curation, Investigation, Methodology, Writing – review & editing. Y-PL: Data curation, Investigation, Methodology, Writing – review & editing. Y-QY: Conceptualization, Data curation, Formal analysis, Funding acquisition, Project administration, Resources, Supervision, Writing – original draft, Writing – review & editing.
